# Supplementary material for: Poly(ADP-ribosyl)ating pathway regulates development from stem cell niche to longevity control
Source: Life Sci Alliance. 2021 Dec 23;5(3):e202101071. doi: 10.26508/lsa.202101071 (PMC8739260; doi:10.26508/lsa.202101071)
Supplement: Supplementary file 1 [file LSA-2021-01071_TableS1.docx]

| Name | Peptide Sequence | Charge | Mass deviation (ppm) | XCorr |
| --- | --- | --- | --- | --- |
| Parg-PA | SET*SAKSS*PELNK | 2 | -1.7 | 2.644 |
| Parg-PA | SETS*AKS*SPELNK | 2 | -1.1 | 3.209 |
| Parg-PA | SETSAKS*S*PELNK | 2 | -2.7 | 3.166 |
| Parg-PA | SETSAKS*S*PELNK | 2 | -0.7 | 3.161 |
| Parg-PA | SETSAKS*S*PELNK | 3 | -0.9 | 1.771 |
| Parg-PA | SETSAKS*S*PELNK | 3 | 0.6 | 1.499 |
| Parg-PA | SPDGGISEIETEEEPENLANS*LDDS*WRGVSMEAIH | 4 | -3.2 | 3.359 |
| Parg-PA | VAGLGEGKS*ETS*AKS*S*PELNK | 3 | 0.4 | 2.297 |
