# Supplementary material for: Poly(ADP-ribosyl)ating pathway regulates development from stem cell niche to longevity control
Source: Life Sci Alliance. 2021 Dec 23;5(3):e202101071. doi: 10.26508/lsa.202101071 (PMC8739260; doi:10.26508/lsa.202101071)
Supplement: Supplementary file 2 [file LSA-2021-01071_TableS2.docx]

Table S2. Subset of Peptide sequences found by mass spectrometry. This experiment has been performed in triplicate (Exp1, Exp2, and Exp3). The score presented in columns 3–5 and 8–10 correspond to the enrichment of the peptide sequence. A score labeled in blue corresponds to a moderately enriched peptide, whereas a purple score corresponds to a highly enriched peptide. The left panel corresponds to unmodified poly(ADP-ribose) glycohydrolase band (lower band of [Fig 2D](#fig2)), whereas the right panel corresponds to the higher band of [Fig 2D](#fig2) (P-PARG*). For each peptide, the phosphorylated residues are represented in red.

| Ref | Peptide Sequence | Exp1 | Exp2 | Exp3 | Ref | Peptide Sequence | Exp1 | EXp2 | Exp3 |
| --- | --- | --- | --- | --- | --- | --- | --- | --- | --- |
| PARG | SE**T**#SAKSSPELNK | 0 | 0 | 0 | PARG* | SE**T**#SAKSSPELNK | 0 | 0 | 0 |
| PARG | SETSAKS**S**#PELNK | 7.37 | 2.21 | 1.95 | PARG* | SETSAKS**S**#PELNK | 0 | 0 | 2 |
| PARG | SE**T**#SAKS**S**#PELNK | 0 | 0 | 0 | PARG* | SE**T**#SAKS**S**#PELNK | 0 | 0 | 0 |
| PARG | SET**S**#AKSSPELNK | 0 | 0 | 0 | PARG* | SET**S**#AKSSPELNK | 1.29 | 2.2 | 1.9 |
| PARG | SETSAK**S**#SPELNK | 11.24 | 21.86 | 1.04 | PARG* | SETSAK**S**#SPELNK | 0 | 0 | 1.2 |
| PARG | SET**S**#AK**S**#SPELNK | 0 | 0 | 1 | PARG* | SET**S**#AK**S**#SPELNK | 13.82 | 24.7 | 19 |
| PARG | SETSAK**S**#SPELNK | 0 | 0 | 0 | PARG* | SETSAK**S**#SPELNK | 4.56 | 3.8 | 1.26 |
| PARG | SETSAKS**S**#PELNK | 3.09 | 17.5 | 4.14 | PARG* | SETSAKS**S**#PELNK | 0 | 0 | 7.31 |
| PARG | SETSAK**S**#**S**#PELNK | 0 | 0 | 1.43 | PARG* | SETSAK**S**#**S**#PELNK | 27.7 | 11.85 | 8.17 |
| PARG | SE**T**#SAKS**S**#PELNK | 0 | 0 | 0 | PARG* | SE**T**#SAKS**S**#PELNK | 0 | 0 | 0 |
| PARG | VAGLGEGK**S**#E**T**#SAKSSPELNK | 0 | 0 | 0 | PARG* | VAGLGEGK**S**#E**T**#SAKSSPELNK | 0 | 0 | 0 |
| PARG | VAGLGEGK**S**#E**T**#SAK**S**#**S**#PELNK | 0 | 0 | 0 | PARG* | VAGLGEGK**S**#E**T**#SAK**S**#**S**#PELNK | 0 | 0 | 0 |
| PARG | VAGLGEGK**S**#ETSAKSSPELNK | 3.96 | 0 | 1.72 | PARG* | VAGLGEGK**S**#ETSAKSSPELNK | 14.8 | 8.8 | 4.93 |
| PARG | VAGLGEGK**S**#ET**S**#AKSSPELNK | 0 | 0 | 0 | PARG* | VAGLGEGK**S**#ET**S**#AKSSPELNK | 7.24 | 2.1 | 4.78 |
| PARG | VAGLGEGK**S**#ETSAK**S**#SPELNK | 0 | 1.21 | 0 | PARG* | VAGLGEGK**S**#ETSAK**S**#SPELNK | 9.81 | 2.66 | 13.20 |
| PARG | VAGLGEGK**S**#ETSAKS**S**#PELNK | 3.15 | 2.81 | 5.21 | PARG* | VAGLGEGK**S**#ETSAKS**S**#PELNK | 2.21 | 0 | 3.76 |
| PARG | VAGLGEGKSETSAK**S**#**S**#PELNK | 0 | 0 | 0 | PARG* | VAGLGEGKSETSAK**S**#**S**#PELNK | 4.29 | 2.29 | 1 |
| PARG | VAGLGEGK**S**#ETSAK**S**#**S**#PELNK | 0 | 0 | 0 | PARG* | VAGLGEGK**S**#ETSAK**S**#**S**#PELNK | 16.78 | 11.56 | 3.45 |
| PARG | VAGLGEGKSET**S**#AK**S**#**S**#PELNK | 0 | 0 | 0 | PARG* | VAGLGEGKSET**S**#AK**S**#**S**#PELNK | 27.09 | 21.47 | 13.37 |
| PARG | VAGLGEGK**S**#ET**S**#AK**S**#**S**#PELNK | 0 | 0 | 0 | PARG* | VAGLGEGK**S**#ET**S**#AK**S**#**S**#PELNK | 67.05 | 43.6 | 38.2 |
| PARG | VAGLGEGK**S**#E**T**#**S**#AK**S**#**S**#PELNK | 0 | 0 | 0 | PARG* | VAGLGEGK**S**#E**T**#**S**#AK**S**#**S**#PELNK | 0 | 0 | 0 |
| PARG | SPDGGISEIETEEEPENLANSLDD**S**#WRGVSMEAIH | 3.18 | 0 | 0 | PARG* | SPDGGISEIETEEEPENLANSLDD**S**#WRGVSMEAIH | 0 | 0 | 0 |
| PARG | SPDGGISEIETEEEPENLAN**S**#LDDSWRGVSMEAIH | 11.74 | 6.31 | 6.61 | PARG* | SPDGGISEIETEEEPENLAN**S**#LDDSWRGVSMEAIH | 7.49 | 11.7 | 10.94 |
| PARG | SPDGGISEIE**T**#EEEPENLAN**S**#LDD**S**#WRGVSMEAIH | 0 | 0 | 0 | PARG* | SPDGGISEIE**T**#EEEPENLAN**S**#LDD**S**#WRGVSMEAIH | 0 | 0 | 0 |
| PARG | SPDGGISEIETEEEPENLAN**S**#LDD**S**#WRGVSMEAIH | 0 | 0 | 0 | PARG* | SPDGGISEIETEEEPENLAN**S**#LDD**S**#WRGVSMEAIH | 39.2 | 93.05 | 36.5 |
| PARG | SPDGGI**S**#EIE**T**#EEEPENLAN**S**#LDD**S**#WRGVSMEAIH | 0 | 0 | 0 | PARG* | SPDGGI**S**#EIE**T**#EEEPENLAN**S**#LDD**S**#WRGVSMEAIH | 0 | 0 | 0 |
| PARG | SPDGGI**S**#EIETEEEPENLANSLDDSWRGVSMEAIH | 0 | 0 | 0 | PARG* | SPDGGI**S**#EIETEEEPENLANSLDDSWRGVSMEAIH | 0 | 0 | 0 |
