# Supplementary material for: Poly(ADP-ribosyl)ating pathway regulates development from stem cell niche to longevity control
Source: Life Sci Alliance. 2021 Dec 23;5(3):e202101071. doi: 10.26508/lsa.202101071 (PMC8739260; doi:10.26508/lsa.202101071)
Supplement: Supplementary file 3 [file LSA-2021-01071_TableS3.docx]

Table S3. Prediction of ph1 and ph2 modification by different kinases using NetPhorest2 software. Peptide sequences corresponding to phosphorylation sites 1 and 2 of *Drosophila* poly(ADP-ribose) glycohydrolase protein were analyzed using NetPhorest2 software [(43)](#bib43) <http://netphorest.info/index.shtml>.

| dPARG AA Position | Peptide | classifier | Posterior Probability | Contributor |
| --- | --- | --- | --- | --- |
| Ser69 | spdggiseieteeepenlan**S**lddswrgvsmeaih | CK2_group | 0.35293 | Phospho.ELM |
|  | spdggiseieteeepenlan**S**lddswrgvsmeaih | NEK1_NEK5_... | 0.21019 | Yaffe Lab |
|  | spdggiseieteeepenlan**S**lddswrgvsmeaih | DMPK_group | 0.19927 | Scansite |
|  | spdggiseieteeepenlan**S**lddswrgvsmeaih | GRK_group | 0.11254 | Phospho.ELM |
|  | spdggiseieteeepenlan**S**lddswrgvsmeaih | GSK3-group | 0.11174 | Phospho.ELM |
|  | spdggiseieteeepenlan**S**lddswrgvsmeaih | PKC-group | 0.06791 | Phospho.ELM |
| Ser73 | spdggiseieteeepenlansldd**S**wrgvsmeaih | NEK1_NEK5_... | 0.21672 | Yaffe Lab |
|  | spdggiseieteeepenlansldd**S**wrgvsmeaih | PKC-group | 0.19971 | Phospho.ELM |
|  | spdggiseieteeepenlansldd**S**wrgvsmeaih | ACTR2_... | 0.11334 | Turk Lab |
|  | spdggiseieteeepenlansldd**S**wrgvsmeaih | DMPK-group | 0.10370 | Scansite |
|  | spdggiseieteeepenlansldd**S**wrgvsmeaih | ROCK-group | 0.06216 | Phospho.ELM |
|  | spdggiseieteeepenlansldd**S**wrgvsmeaih | CK2_group | 0.01753 | Phospho.ELM |
| Ser621 | vaglgegk**S**etsaksspelnk | CK2_group | 0.20078 | Phospho.ELM |
|  | vaglgegk**S**etsaksspelnk | ACTR2_... | 0.08904 | Turk Lab |
|  | vaglgegk**S**etsaksspelnk | PKC_group | 0.08862 | Phospho.ELM |
|  | vaglgegk**S**etsaksspelnk | PKD_group | 0.06321 | Scansite |
|  | vaglgegk**S**etsaksspelnk | AMPK_group | 0.05377 | Scansite |
|  | vaglgegk**S**etsaksspelnk | NEK1_NEK5_... | 0.05133 | Yaffe Lab |
| Thr623 | vaglgegkse**T**saksspelnk | ACTR2_... | 0.21937 | Turk Lab |
|  | vaglgegkse**T**saksspelnk | PAKB_group | 0.13154 | Turk Lab |
|  | vaglgegkse**T**saksspelnk | PKC_group | 0.11862 | Phospho.ELM |
|  | vaglgegkse**T**saksspelnk | GSK3-group | 0.11026 | Yaffe Lab |
|  | vaglgegkse**T**saksspelnk | MAPKAPK_... | 0.08208 | Phospho.ELM |
|  | vaglgegkse**T**saksspelnk | CK2_group | 0.05748 | Phospho.ELM |
| Ser624 | vaglgegkset**S**aksspelnk | ACTR2_... | 0.21228 | Turk Lab |
|  | vaglgegkset**S**aksspelnk | PKC_group | 0.17501 | Phospho.ELM |
|  | vaglgegkset**S**aksspelnk | GSK3-group | 0.15711 | Yaffe Lab |
|  | vaglgegkset**S**aksspelnk | CK1-group | 0.11274 | Scansite |
|  | vaglgegkset**S**aksspelnk | NEK1_NEK5_... | 0.08044 | Yaffe Lab |
|  | vaglgegkset**S**aksspelnk | CK2_group | 0.04356 | Phospho.ELM |
| Ser627 | vaglgegksetsak**S**spelnk | CK2_group | 0.44361 | Phospho.ELM |
|  | vaglgegksetsak**S**spelnk | NEK1_NEK5_... | 0.13974 | Yaffe Lab |
|  | vaglgegksetsak**S**spelnk | CK1-group | 0.13515 | Scansite |
|  | vaglgegksetsak**S**spelnk | PKC_group | 0.10298 | Phospho.ELM |
|  | vaglgegksetsak**S**spelnk | GSK3-group | 0.08867 | Phospho.ELM |
|  | vaglgegksetsak**S**spelnk | ACTR2_... | 0.08432 | Turk Lab |
| Ser628 | vaglgegksetsaks**S**pelnk | p38_group | 0.17496 | Scansite |
|  | vaglgegksetsaks**S**pelnk | MAPK3_... | 0.11258 | Phospho.ELM |
|  | vaglgegksetsaks**S**pelnk | CDK5 | 0.08403 | Scansite |
|  | vaglgegksetsaks**S**pelnk | GSK3-group | 0.08316 | Phospho.ELM |
|  | vaglgegksetsaks**S**pelnk | NEK1_NEK5_... | 0.03791 | Yaffe Lab |
|  | vaglgegksetsaks**S**pelnk | CK2_group | 0.00765 | Phospho.ELM |
